# Peripheral inflammation is associated with remote global gene expression changes in the brain

**DOI:** 10.1186/1742-2094-11-73

**Published:** 2014-04-08

**Authors:** Carolyn A Thomson, Alison McColl, Jonathan Cavanagh, Gerard J Graham

**Affiliations:** 1 Institute of Infection, Immunity & Inflammation, College of Medical & Veterinary Life Sciences, University of Glasgow, 120 University Place, Glasgow G12 8TA, UK; 2 Institute of Health & Wellbeing, College of Medical & Veterinary Life Sciences, University of Glasgow, Southern General Hospital, Glasgow G51 4TF, UK

**Keywords:** cytokines, innate immunity, interferons, interferon-stimulated genes, lipopolysaccharide, neuroimmune communication, neuroinflammation, systemic inflammation, toll-like-receptor ligation

## Abstract

**Background:**

Although the central nervous system (CNS) was once considered an immunologically privileged site, in recent years it has become increasingly evident that cross talk between the immune system and the CNS does occur. As a result, patients with chronic inflammatory diseases, such as rheumatoid arthritis, inflammatory bowel disease or psoriasis, are often further burdened with neuropsychiatric symptoms, such as depression, anxiety and fatigue. Despite the recent advances in our understanding of neuroimmune communication pathways, the precise effect of peripheral immune activation on neural circuitry remains unclear. Utilizing transcriptomics in a well-characterized murine model of systemic inflammation, we have started to investigate the molecular mechanisms by which inflammation originating in the periphery can induce transcriptional modulation in the brain.

**Methods:**

Several different systemic and tissue-specific models of peripheral toll-like-receptor-(TLR)-driven (lipopolysaccharide (LPS), lipoteichoic acid and Imiquimod) and sterile (tumour necrosis factor (TNF) and 12-O-tetradecanoylphorbol-13-acetate (TPA)) inflammation were induced in C57BL/6 mice. Whole brain transcriptional profiles were assessed and compared 48 hours after intraperitoneal injection of lipopolysaccharide or vehicle, using Affymetrix GeneChip microarrays. Target gene induction, identified by microarray analysis, was validated independently using qPCR. Expression of the same panel of target genes was then investigated in a number of sterile and other TLR-dependent models of peripheral inflammation.

**Results:**

Microarray analysis of whole brains collected 48 hr after LPS challenge revealed increased transcription of a range of interferon-stimulated genes (ISGs) in the brain. In addition to acute LPS challenge, ISGs were induced in the brain following both chronic LPS-induced systemic inflammation and Imiquimod-induced skin inflammation. Unique to the brain, this transcriptional response is indicative of peripherally triggered, interferon-mediated CNS inflammation. Similar models of sterile inflammation and lipoteichoic-acid-induced systemic inflammation did not share the capacity to trigger ISG induction in the brain.

**Conclusions:**

These data highlight ISG induction in the brain as being a consequence of a TLR-induced type I interferon response. As considerable evidence links type I interferons to psychiatric disorders, we hypothesize that interferon production in the brain could represent an important mechanism, linking peripheral TLR-induced inflammation with behavioural changes.

## Background

By mechanisms that remain to be fully established, systemic infection or inflammation can have a profound effect on the central nervous system (CNS), manifesting in a number of behavioural adaptations, as well as fever and increased neuroendocrine activation. Promoting energy conservation and minimizing heat loss, these sickness behaviours represent a sound strategy designed to help an organism overcome infection. Symptoms include fever, malaise, anorexia, lethargy and, in severe cases, neuropsychiatric disorders, such as depression and anxiety [1]. Sickness behaviours occur during acute bacterial or viral infections, but also during chronic inflammatory diseases, such as rheumatoid arthritis, inflammatory bowel disease and psoriasis [2-4]. In the case of the latter, what would be a beneficial, self-limiting, system can become dysregulated. The prolonged depression and anxiety that ensues represents a major burden to patients, not least because these detrimental comorbidities lead to a poorer clinical outcome.

It is becoming increasingly evident that sickness behaviours are triggered as a result of biological, inflammatory, pathways. In particular, inflammatory cytokines, such as tumour necrosis factor α (TNFα), interleukin-1 β (IL-1β) and interleukin-6 (IL-6) play a pivotal role in inducing symptoms of sickness behaviour. Although not a prerequisite for any of the psychiatric symptoms, IL-6 is required for the induction of a fever response [5], whereas behavioural changes are thought to be attributable to IL-1β and TNFα. Using animal models, it has been demonstrated that most behavioural symptoms can be induced by peripheral, or central, administration of either IL-1β or TNFα [6,7]. Furthermore, several cytokines have been implicated in the manifestation of major depressive disorders in patients with chronic inflammatory diseases. For example, in a phase III clinical trial in which patients with moderate-to-severe psoriasis were treated with the soluble TNFα receptor etanercept, improvement in depression scores preceded the improvements seen in terms of psoriasis severity [4]. Supporting the notion of cytokine-induced depression, patients receiving interferon (IFN) α or IFNβ therapy face a risk of experiencing depression as a side effect of treatment [8-12]. Moreover, patients with major depressive disorders, and no clinical signs of inflammation, often present with elevated levels of circulating inflammatory cytokines [13]. Therefore, significant quantities of literature back the immune system, in particular inflammatory cytokine production, as a key contributor to sickness-induced behavioural changes.

Once considered an immunologically privileged site, the CNS is well fortified against changes in the periphery. However, cross talk does occur and, as a result, much research has gone into elucidating putative routes of immune-to-brain communication. In spite of this, the precise effect of peripheral immune activation on neural circuitry remains unclear. With the aim of better unravelling neuroimmune communication pathways, and the downstream consequences of peripheral inflammation on the brain, we compared gene expression in the brains of mice following several different sterile (tumour necrosis factor (TNF) and 12-O-tetradecanoylphorbol-13-acetate (TPA) and toll-like-receptor-(TLR)-dependent (lipopolysaccharide (LPS), lipoteichoic acid (LTA) and Imiquimod) models of peripheral inflammation. We also compared gene expression in the brain with that of peripheral blood leucocytes (PBLs). Lastly, we explored a potential molecular mechanism by which inflammation originating in the periphery can induce transcriptional modulation in the brain.

## Methods

### Mice

Wild type C57BL/6 mice (7 to 8 weeks old, 20 to 25 g) were purchased from Harlan Laboratories. Mice were maintained in specific pathogen-free conditions in standard caging in the Central Research Facility at the University of Glasgow and treated with sterile (TNF and TPA) and TLR ligand-based (LPS, LTA and Imiquimod) inflammatory agents as described. For microarray experiments, a minimum of three biological replicates are required to allow statistical analysis of the data [14]. Three mice were used per study arm for all our microarray experiments and four or five mice each for qPCR-based experiments, to ensure the statistical robustness of the data. All experiments received ethical approval and were performed under the auspices of UK Home Office Licences.

### Acute inflammatory models

For acute LPS-induced inflammation, mice were injected intraperitoneally (i.p.) with 100 μl of 1 mg/ml LPS (≈4 mg/kg), derived from *Escherichia coli* serotype 055:B5 (Sigma, St. Louis, MO, USA, or an equivalent volume of vehicle (PBS). For TNFα- or LTA-induced inflammation, mice were injected intravenously (i.v.) with two doses of 1 μg recombinant TNFα (Peprotech, Rocky Hill, NJ, USA), two doses of 500 μg LTA (Sigma, St. Louis, MO, USA) or two doses of an equivalent volume (100 μl) of vehicle (sterile H_2_O) at 0 and 24 hours. Mice were euthanized by CO_2_ exposure 48 hours after initial injection and perfused for 5 minutes with 20 ml PBS.

### Chronic inflammatory models

For chronic LPS-induced inflammation, and induction of endotoxin tolerance, mice received a daily i.p. injection of 100 μl of 0.5 mg/ml LPS (≈2 mg/kg) (Sigma, St. Louis, MO, USA) or an equivalent volume (100 μl) of vehicle (PBS) for 2, 5 or 7 consecutive days. For skin-inflammation models, mice were shaved on their dorsal skin 24 hours prior to receiving daily applications of ≈ 80 mg of 5% Imiquimod (Aldara™, MEDA Ab, Stockholm, Sweden) cream [15], 150 μl of 100 μM TPA, or an equivalent volume of Vaseline (Unilever, Leatherhead, UK) or acetone control. Mice were treated for 5 consecutive days as described previously [15]. All mice were euthanized by CO_2_ exposure 24 hours after final treatment and perfused for 5 minutes with 20 ml PBS.

### ELISA

Blood was collected from tail veins (approximately 300 μl) prior to termination of the mice. Plasma was isolated from whole blood by centrifugation. Throughout the study, plasma concentrations of soluble mediators, IL-1β, TNFα and IL-6, were determined using DuoSet ELISA kits (R&D Systems, Minneapolis, MN, USA) according to the manufacturer’s instructions.

### RNA isolation from tissue and peripheral blood leucocytes

Whole brain tissue was snap frozen and stored at -80°C until use. Under RNase-free conditions, brains were homogenized using the TissueLyser LT (Qiagen, Hilden, Germany). RNA was extracted from homogenized tissue using Trizol® (Life Technologies, Invitrogen, Carlsbad, CA, USA) as described by the manufacturers. Isolated RNA was further purified and genomic DNA removed using an RNeasy Mini Kit (Qiagen, Hilden, Germany). Red blood cells were lysed from blood samples using red blood cell lysis buffer (Miltenyi, Cologne, Germany). Under RNase-free conditions, RNA was isolated and genomic DNA was removed from PBLs using an RNeasy Micro Kit (Qiagen, Hilden, Germany).

### GeneChip microarray analysis

Microarray assays were performed in the Glasgow Polyomics Facility at the University of Glasgow [16]. Briefly, 1 μg of purified total RNA was amplified by *in-vitro* transcription and converted to sense-strand cDNA using a WT Expression kit (Life Technologies, Invitrogen, Carlsbad, CA, USA). cDNA was then fragmented and labelled using a GeneChip WT Terminal Labelling kit (Affymetrix, Santa Clara, CA, USA). Fragmented cDNA samples were then hybridized to GeneChip Mouse Gene 1.0 ST Arrays (Affymetrix, Santa Clara, CA, USA). Procedures were carried out as described by the manufacturers.

To maximize the identification of key differentially expressed genes we utilized two separate software analysis packages (Partek and GeneSpring) and focused on gene expression differences identified using both approaches. As shown in the results section, this reduced the number of genes requiring analysis and provided increased confidence in their validity.

Data generated using Partek Genomics Suite were normalized using the robust multichip average (RMA) method, adjusted for GC content. The normalized data were subsequently analyzed using one-way analysis of variance (ANOVA) to determine the significance of each gene in LPS-treated mice compared with vehicle-treated controls. Data generated using GeneSpring GX software were normalized using RMA 16. Normalized data were analyzed using unpaired *t* tests to determine the significance of gene expression differences in LPS-treated mice compared with vehicle-treated controls. In both analyses, *P* values were adjusted for multiple comparisons using the Benjamini-Hochberg multiple testing correction.

Gene ontology terms were assigned to differentially expressed genes using the Database for Annotation, Visualization and Integrated Discovery (DAVID) Bioinformatics Resources v6.7 [17]. Analysis was performed in accordance with two protocols outlined by Huang *et al.*[18,19]. Significance of enrichment was determined using a modified Fisher’s exact test and a Benjamini-Hochberg multiple testing correction was used to correct for the rate of type I errors. Co-expression of a gene cluster was considered significant for *P* ≤ 0.05.

Genes were grouped into canonical pathways using Ingenuity Pathway Analysis software (Ingenuity® Systems [20]). Significance of differentially altered pathways was determined using a Fisher’s exact test and a Benjamini-Hochberg multiple testing correction was used to correct for the rate of type I errors. Enrichment of a pathway was considered significant for *P* ≤ 0.05.

### qPCR

Total RNA was reverse transcribed using Quantitect® Reverse Transcription kit (Qiagen, Hilden, Germany) using random primers. Quantitative real-time PCR (QPCR) amplifications were performed in triplicate using PerfeCTa® SYBR® Green FastMix® (Quanta Biosystems, Gaithersburg, MD, USA). A 500 μM mix of forward and reverse primers was used per reaction. Primers were designed using Primer3 Input software (version 0.4.0) and generated by IDT technologies. Primer sequences are listed in Additional file 1: Table S1. qPCR reactions were performed using a Prism® 7500HT Sequence Detection System (Life Technologies, Invitrogen, CA, USA) for 40 cycles, in accordance with the manufacturer’s guidelines. The absolute copy number was calculated from a standard curve and normalized to a reference gene, TATA binding protein (TBP), as previously described [21]. Fold change values were calculated by comparing the normalized copy number of individual samples with the mean of the control samples.

### Histology

Skin samples were fixed in 10% buffered formalin prior to processing and paraffin embedding. Processing was performed using the Shandon Citadel 1000 automated tissue processor (Thermo scientific). Embedded tissue was then cut into 5 μm sections and stained with (H & E) to discern morphology.

## Results

### Peripheral LPS challenge triggers an inflammatory response in the brain

**Figure 1:**
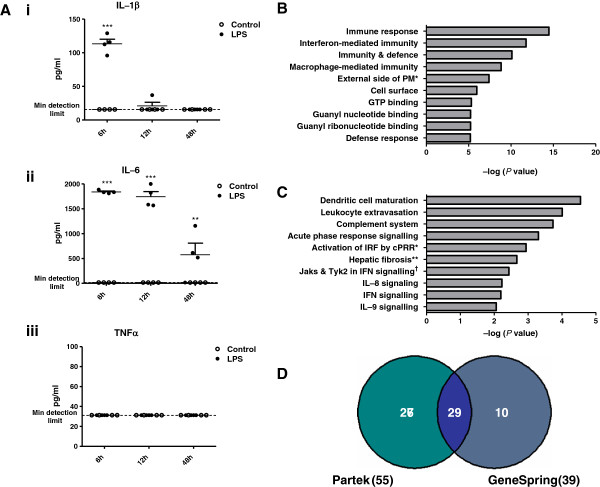
**Systemic LPS injection results in an inflammatory response in the periphery and brain. (A)** Plasma concentrations of (i) IL-1β, (ii) IL-6 and (iii) TNFα, 6, 12 and 48 hours following injection with LPS (100 μg i.p.) or vehicle. Data represent the mean ± the standard error of the mean (SEM). Significance was determined using two-way ANOVA: **P ≤ 0.01, ***P ≤ 0.001; *n* = 4/group. **(B)** Top enriched gene ontology classifications determined using DAVID Bioinformatics Resources. **(C)** Top significantly altered pathways determined using Ingenuity Pathway Analysis software. **(B,C)** Significance was calculated using Fisher’s exact test. **(D)** Comparison of entities identified using Partek Genomics Suite and Genespring GX software packages as being differentially expressed by ≥ 2-fold.

To induce systemic inflammation, mice were injected with a single high dose of LPS (100 μg i.p.); 6 hours following injection, plasma from all mice injected with LPS displayed significantly elevated levels of IL-1β and IL-6 (Figure 1A). Although TNFα was not elevated, it has previously been shown that TNFα levels peak in the circulation of C57BL/6 mice 2 hours following LPS injection (100 μg i.p.) and then rapidly decline [22]. Levels of IL-1β returned to baseline between 6 and 12 hours following injection and IL-6 levels remained elevated until 48 hours. Thus, systemic LPS injection triggers a potent and rapid inflammatory response in the periphery.

Systemic administration of high doses of LPS is known to activate the innate immune system in the CNS, occurring throughout the circumventricular organs, the meninges and the parenchyma [23,24]. To determine what downstream effects this has on global gene expression throughout the brain, Affymetrix GeneChip microarrays were used to compare transcriptional profiles of brain tissue 48 hours after LPS or vehicle injection. The time point of 48 hours was selected to allow time for the transcription and translation of peripheral responses to LPS and the subsequent initiation of transcriptional responses in the brain. Whilst it is not possible to define rigorously when these processes will have been completed, 48 hours was selected as a plausible time point. All mice were perfused extensively (20 ml over 5 minutes) with PBS to remove contaminating peripheral blood from the harvested brains. When analyzed using Partek Genomics Suite, 85 entities were differentially expressed in the brains by at least 1.5-fold (Additional file 2: Table S2). Therefore, 48 hours after challenge, the transcriptional profile of the brain is altered in response to systemic LPS-induced inflammation.

Differentially expressed genes with a fold change value of 1.5 or greater were subjected to gene ontology clustering using the DAVID Bioinformatics Resources. Amongst the most significantly enriched biological processes are: ‘immune response’, ‘immunity and defence’, ‘macrophage-mediated immunity’ and ‘interferon-mediated immunity’ (Figure 1B). This strongly implies the presence of a remotely triggered immune or inflammatory response in the brain.

To complement and validate the data generated using DAVID, the putative interactions and functional relationships between the protein products of the significantly altered genes were assessed using Ingenuity Pathway Analysis software. Significantly altered pathways included: ‘activation of interferon regulatory factors (IRFs) by cytosolic pattern recognition receptors (cPRRs)’, ‘Jak1, Jak2 and Tyk2 in interferon signalling’ and ‘interferon signalling’ (Figure 1C). The transcriptional enhancement of IFN signalling pathway components, coupled with the enrichment of genes involved in IFN-mediated immunity, suggests that systemic LPS challenge might induce an IFN response in the brain.

Analyzing the Affymetrix dataset using Partek Genomics Suite returned a list of 55 differentially expressed entities that satisfied a more stringent fold change cut-off of 2 (Additional file 2: Table S2). Prior to qPCR validation, with the aim of focusing only on the genes that are most robustly differentially expressed, the Affymetrix dataset was reanalyzed using GeneSpring GX analysis software, and the resulting list of significantly regulated genes was compared with that generated using Partek. The 39 entities identified by GeneSpring as being upregulated by at least 2-fold (Additional file 3: Table S3) were then compared with those identified using Partek (Figure 1D). Of the 29 entities that were common to both lists, 24 are known genes. These are grouped according to biological function in Table 1. Strikingly, over half of the genes in this list are interferon-stimulated genes (ISGs). This supports the hypothesis generated from the Ingenuity Pathway analysis; that IFN signalling is induced in the brain following peripheral LPS injection. As they have been simultaneously validated using two bioinformatics approaches, ISGs from this sub-list of genes (Table 1) have subsequently been focused on for the remainder of the study, with the assumption that these genes are most likely to be biologically relevant.

**Table 1:** Functional properties of differentially expressed target genes

**Gene symbol**	**Gene name**
**Interferon-stimulated genes**	
*Ctsc*	Cathepsin C
*Gbp2*	Guanylate-binding protein 2
*Gbp3*	Guanylate-binding protein 3
*Gbp6*	Guanylate-binding protein 6
*Gbp7*	Guanylate-binding protein 7
*Ifit1*	Interferon-induced protein with tetratricopeptide repeats
*Ifitm3*	Interferon-induced transmembrane protein
*Irgm1*	Immunity-related GTPase, family M, member 1
*Lgals3bp*	Lectin, galactoside-binding, soluble, 3 binding protein
*Oasl2*	2′-5′ Oligoadenylate synthetase-like 2
*Rnf213*	Ring finger protein 213
*Rtp4*	Receptor transporter protein 4
*Sp100*	Nuclear antigen Sp100
*Stat1*	Signal transducer and activator of transcription 1
**Acute phase reactants**	
*Lcn2*	Lipocalin 2
*Saa3*	Serum amyloid A3
*Serpina3n*	Serine (or cysteine) peptidase inhibitor, clade A
**Immunity system components**	
*C4b |C4a*	Complement component 4B (Childo blood group) | complement 4A (Rodgers blood group)
*Fcgr4*	Fc receptor, IgG, low affinity IV
*H2-K1*	Histocompatibility 2, K1, K region
*Pglyrp1*	Peptidoglycan recognition protein 1
**Cell surface molecules**	
*Il2rg*	Interleukin 2 receptor, gamma chain
*Ly6a*	Lymphocyte antigen 6 complex, locus A
**mRNA editing**	
*Apobec3*	Apolipoprotein B mRNA editing enzyme

### Differential expression of ISGs was confirmed using qPCR

**Figure 2:**
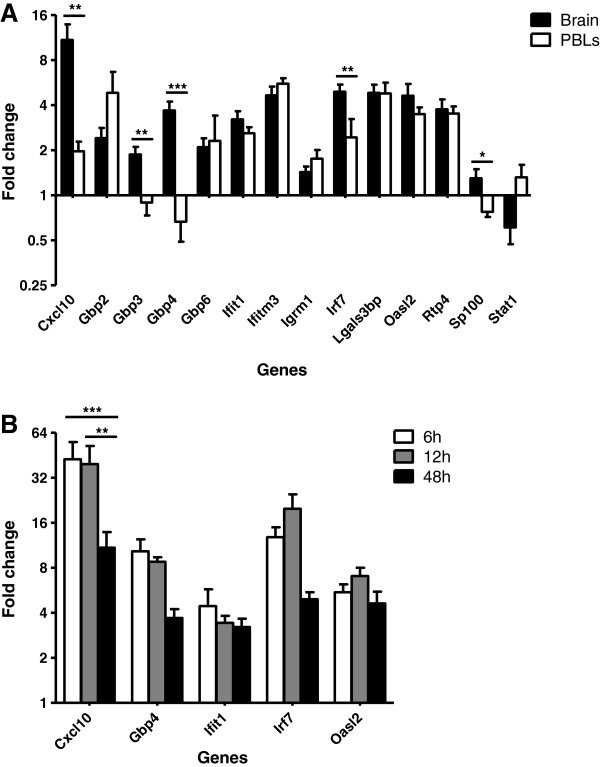
**ISG induction in the brain and blood of LPS-treated mice. (A)** Relative expression of ISGs in the brain and PBLs, 48 hours after LPS injection (100 μg i.p.) compared with vehicle injection. **(B)** Relative expression of ISGs in the brain, 6, 12 and 48 hours after LPS injection compared with vehicle injection. Gene expression normalized to TBP. Fold change calculated by comparing the normalized copy number in each sample with the mean of the vehicle-injected controls. Data represent mean ± SEM. Statistical significance was determined using two-way ANOVA: *P ≤ 0.05, **P ≤ 0.01, ***P ≤ 0.001; *n* = 4/group.

Using RNA isolated from an independent experiment, ISG upregulation in the brain following LPS injection was then validated using qPCR. Even though brains were perfused, to control for the possibility of a residual contaminating signal coming from the peripheral blood, the expression of ISGs was compared in the brain and PBLs. In addition to target ISGs (Table 1), upregulation of genes encoding the classic interferon-inducible chemokine CXCL10, and IRF7, were also validated by qPCR, as was the upregulation of the gene encoding the negative regulator of IRF7, guanylate-binding protein (GBP) 4. Owing to the relatively high proportion of GBPs in the dataset, *Gbp6* and *Gbp7* were arbitrarily excluded from validation. With the exception of *Sp100* and *Stat1*, all ISGs assayed were significantly upregulated in the brains of mice challenged with systemic LPS compared with vehicle controls (Figure 2A, Table 2). Although many of the genes were upregulated to a similar extent in both the brain and PBLs, in terms of fold change, *Cxcl10*, *Irf7*, *Gbp3* and *Gbp4* were upregulated independently in the brain. Thus, our data demonstrate a differential pattern of gene expression in the brain from that of the PBLs. Not only do these crucial observations provide evidence of a brain-specific inflammatory response as a result of systemic LPS injection, but they support the validity of the dataset; confirming an upregulation of target genes whilst simultaneously verifying that the observed fold changes in the brain are not a secondary effect of blood contamination.

**Table 2:** Significance of differentially expressed target genes following acute inflammatory models

**Gene**	**Significance of fold change in treatment group compared to vehicle control group (↑, upregulated; ↓, downregulated)**					
	**LPS (*P*)**		**TNFα (*P*)**		**LTA (*P*)**	
	**Brain**	**PBLs**	**Brain**	**PBLs**	**Brain**	**PBLs**
***Ctsc***	↑ 0.0332	↑ 0.0283	Not significant	Not significant	Not significant	Not significant
***Cxcl10***	↑ 0.0122	↑ 0.0329	Not significant	↓ 0.0016	Not significant	Not significant
***Gbp2***	↑ 0.0160	Not significant	Not significant	↓ 0.0288	Not significant	Not significant
***Gbp3***	↑ 0.0197	Not significant	Not significant	↓ 0.0179	Not significant	Not significant
***Gbp4***	↑ 0.0030	Not significant	↑ 0.0334	↓ 0.0478	Not significant	Not significant
***Ifit1***	↑ 0.0027	↑ 0.0010	Not significant	Not significant	Not significant	Not significant
***Ifitm3***	↑ 0.0016	↑ 0.0001	Not significant	Not significant	Not significant	↑ 0.0481
***Igrm1***	↑ 0.0343	↑ 0.0311	Not significant	Not significant	Not significant	Not significant
***Irf7***	↑ 0.0004	Not significant	Not significant	Not significant	Not significant	Not significant
***Lgals3bp***	↑ 0.0012	↑ 0.0055	Not significant	Not significant	Not significant	↑ 0.0030
***Oasl2***	↑ 0.0075	↑ 0.0008	Not significant	Not significant	Not significant	Not significant
***Rtp4***	↑ 0.0044	↑ 0.0010	Not significant	Not significant	Not significant	Not significant
***Sp100***	Not significant	↓ 0.0101	Not significant	↓ 0.0062	Not significant	Not significant
***Stat1***	Not significant	Not significant	↑ 0.0252	↓ 0.0371	Not significant	Not significant

Significance of differential gene expression was calculated separately for individual genes in each tissue using an unpaired *t* test.

### ISGs are rapidly induced in the brain following systemic LPS injection

In the immediate hours following a single injection of LPS, the response in the brain is well characterized. Largely encompassing an acute phase response, the majority of reported effects in the brain are known to peak within 12 hours following peripheral LPS injection [24-26]. To determine whether the upregulation of ISGs is a remnant of an earlier response, we looked at expression levels of a selection of ISGs in the brain 6 hours and 12 hours following injection and compared them with the expression levels at 48 hours (Figure 2B). With the exception of *Cxcl10*, the expression levels of which are significantly reduced at 48 hours, no significant differences were observed in the expression levels of ISGs at 48 hours, compared with either 6 hours or 12 hours. Thus, ISGs are induced in the brain by 6 hours following systemic LPS injection and this response persists until 48 hours after injection.

### ISGs were not induced in the brain following peripheral TNFα-induced inflammation

**Figure 3:**
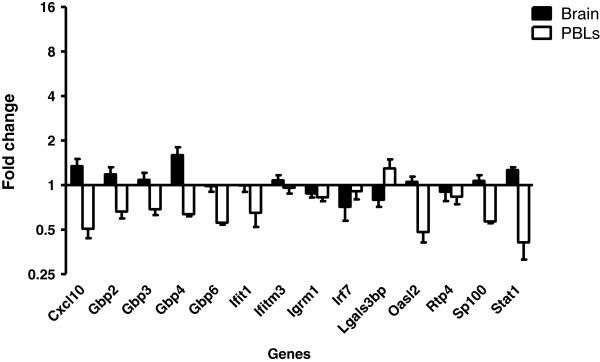
**Response in the brain to systemic administration of TNF.** Mice were injected with two doses of TNFα (1 μg i.v.) or vehicle at 0 and 24 hours and relative expression of interferon-stimulated genes (ISGs) in the brain and peripheral blood leucocytes (PBLs) was determined 48 hours after initial injection. Gene expression was normalized to TBP. Fold change was calculated by comparing the normalized copy number in each sample with the mean of the vehicle-injected control group. Data represent mean ± SEM.

One of the accepted routes of brain sensitization following systemic LPS challenge is the activation of endothelial cells in the brain vasculature by circulating cytokines [26]. To determine whether circulating cytokines were sufficient to induce the expression of ISGs in the brain, mice were challenged with recombinant murine TNFα (1 μg i.v. at 0 and 24 hours). After 48 hours, the expression of the target ISGs was quantified by qPCR. *Gbp4* and *Stat1* were the only ISGs to show slight but significant elevation (1.59- and 1.26-fold, respectively) in the brain following TNFα-injection (Figure 3, Table 2). In fact, several ISGs were significantly downregulated in matched PBLs. Given that the concentrations of systemic TNFα that would result from the intravenous injection of 1 μg would be considerably higher than those induced by systemic LPS (levels were undetectable, as shown in Figure 1), these data collectively suggest that central ISG induction following LPS challenge cannot be accounted for simply on the basis of elevated TNFα in the circulation.

### Inflammatory cytokines and ISGs remain elevated in the brain during endotoxin tolerance

Although ISGs were not induced in the brain in response to circulating TNFα, the previous experiment did not rule out the contribution of other inflammatory cytokines. With the aim of establishing the temporal pattern of ISG expression in the brain and PBLs in the context of endotoxin tolerance, when inflammatory cytokine production is known to be ameliorated in the periphery, we injected mice with a single dose of LPS (50 μg i.p.) daily for 2, 5 or 7 consecutive days. This contrasts with the use of a single application of 100 μg for the initiation of acute LPS effects. Endotoxin tolerance is an important defence mechanism designed to protect the host against endotoxic shock. In addition to eliciting expression of a number of proinflammatory genes, initial cellular exposure to LPS triggers the simultaneous downregulation of TLR expression and the induction of several inhibitory molecules that negatively regulate TLR signalling [27]. This culminates in a transient inactivation of various proinflammatory genes by leucocytes in the periphery, including genes encoding the inflammatory cytokines TNFα, IL-1β and IL-6. At the same time, less potentially pathogenic genes are primed, such as antimicrobial effectors [28]. As a consequence, repeated exposure to TLR ligands leads to a dampening of the proinflammatory milieu without compromising host defence. Conversely, cytokine expression has been shown to continue in the brain during endotoxin tolerance [29,30].

**Figure 4:**
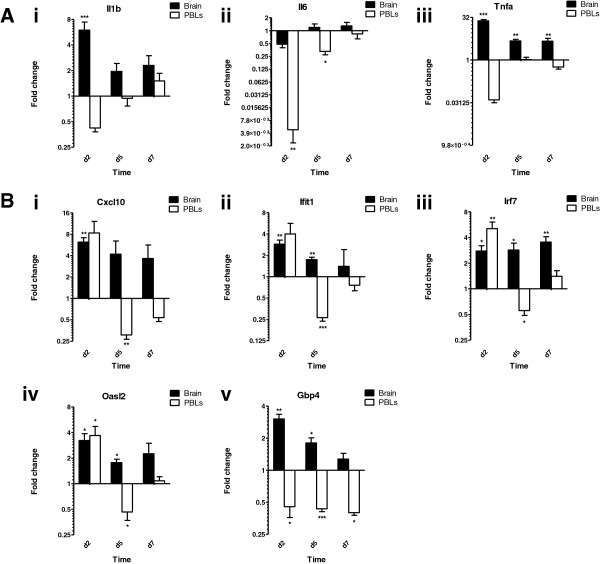
**ISG induction in the brain and PBL of mice treated daily with LPS.** Mice were injected daily with LPS (50 μg i.p.) or vehicle. **(A,B)** Relative gene expression in the brains and PBLs of mice, 2, 5 and 7 days following initial injection. **(A)** Relative expression of cytokines; (i) Il1b, (ii) Il6 and (iii) Tnfa. **(B)** Relative expression of target ISGs; (i) Cxcl10, (ii) Ifit1, (iii) Irf7, (iv) Oasl2 and (v) Gbp4. Gene expression normalized to TBP. Fold change was calculated by comparing the normalized copy number in each sample with the mean of the vehicle-injected controls. Data represent mean ± SEM. Statistical significance was determined using two-way ANOVA: *P ≤ 0.05, **P ≤ 0.01, ***P ≤ 0.001; *n* = 4/group.

Consistent with previous reports, *Il1b* and *Tnfa* were independently upregulated in the brain following multiple LPS challenges (Figure 4A). As anticipated, under these conditions, inflammatory cytokine transcripts were not induced in PBLs. Interestingly, whilst ISG expression is rapidly dampened in the periphery, expression continues in the brain, gradually decreasing between days 2 and 7 (Figure 4B). At day 2, all ISGs were significantly upregulated in the brains of the LPS-challenged mice. At the same time point, most ISGs were similarly upregulated in PBLs as in brain, with the exception of *GBP4*, which was downregulated. On day 5, ISGs remained induced in the brain. All but *Cxcl10* were significantly upregulated at this time point. In contrast, the same genes were significantly downregulated by PBLs. By day 7, ISG transcript levels began to return to baseline, with the exception of *Irf7*, which remained significantly induced. Thus, in comparison with PBLs, it would appear that the brain exhibits a specific and prolonged response to repeated LPS challenge. Not only does this further suggests that LPS-induced ISG expression in the brain is unlikely to be a downstream consequence of peripheral inflammatory cytokines, it again suggests differential mechanism of gene regulation in the brain and the PBLs.

### Systemic administration of LTA was unable to induce an IFN response in the brain

**Figure 5:**
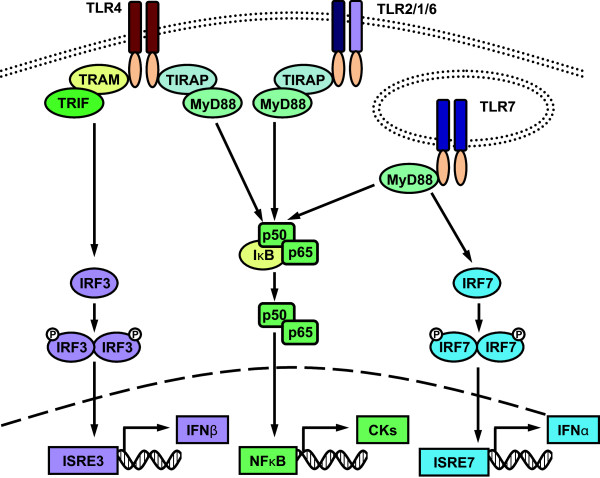
**TLR-induced signalling pathways.** When activated, toll-like receptors (TLRs) either heterodimerize, such as TLR2 with either TLR1 or TLR6, or homodimerize to induce signalling pathway activation. Signalling is regulated by adaptor molecules. MyD88 is an adaptor used by all TLRs aside from TLR3. TLR7 recruits MyD88 directly, whereas TLR4 and TLR2 recruit MyD88 through bridging adapter TIRAP. MyD88 recruitment ultimately leads to the activation of nuclear transcription factor NFκB by releasing the p50 and p65 subunits from the NFκB inhibitor IκB. Activated NFκB then translocates to the nucleus to induce the expression of inflammatory cytokines. When activated by TLR7, MyD88 can also induce the phosphorylation and dimerization of interferon regulatory factor 7 (IRF7). This activation of IRF7 triggers the induction of IFNα. An alternative signalling pathway downstream of TLR4 involves the recruitment of adaptor molecule TRIF through bridging protein TRAM. This ultimately leads to activation of IRF3 and subsequent IFNβ induction (For review see [31]). Note that for simplicity the full complement of extracellular proteins involved in TLR for function are not represented in this diagram. Specifically, TLR4 requires MD-2, CD14 and LPS binding protein to bind LPS efficiently.

**Figure 6:**
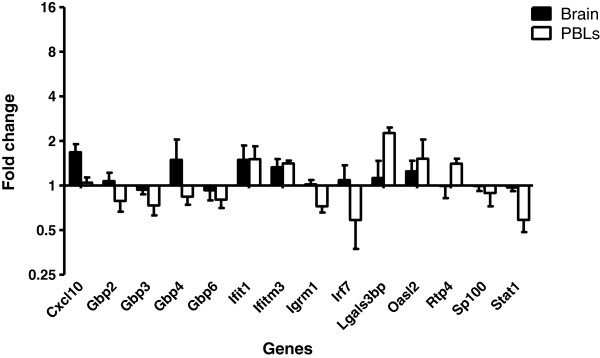
**Response in the brain to systemic administration of LTA.** Mice were injected with two doses of LTA (500 μg i.v.) or vehicle at 0 and 24 hours and relative expression of interferon-stimulated genes (ISGs) in the brain and peripheral blood leucocytes (PBLs) was determined 48 hours after initial injection. Gene expression was normalized to TBP. Fold change was calculated by comparing the normalized copy number in each sample with the mean of the vehicle-injected control group. Data represent mean ± SEM; *n* = 4/group.

LPS binds to TLR4 and exerts its effects via one of two downstream signalling pathways (Figure 5): activation of NFκB through the classical MyD88-dependent pathway, which results in inflammatory cytokine induction, or IRF3 activation through the domain-containing-adaptor-protein-(TRIF)-dependent pathway, which triggers IFNβ production [31]. As many of the genes upregulated in response to LPS can be regulated by IFNs, it is possible that they are being expressed downstream of LPS-induced IFNβ production. To further investigate this possibility, mice were challenged systemically with two high doses of the TLR2 ligand, LTA (500 μg i.v. at 0 and 24 hours). TLR2 ligation can only activate NFκB through the classical pathway [31], thereby eliminating the possibility of TRIF-dependent signalling (Figure 5). As we hypothesized, no significant ISG induction was detected in the brain following LTA-injections (Figure 6, Table 2). *Ifitm3* and *Lgals3bp* were the only ISGs to be significantly induced in PBL. Consequently, activation of the MyD88-dependent pathway alone is insufficient to mimic the brain inflammation that occurs following systemic LPS challenge.

### Brain-specific ISG induction is seen following induction of psoriasis-like skin inflammation

**Figure 7:**
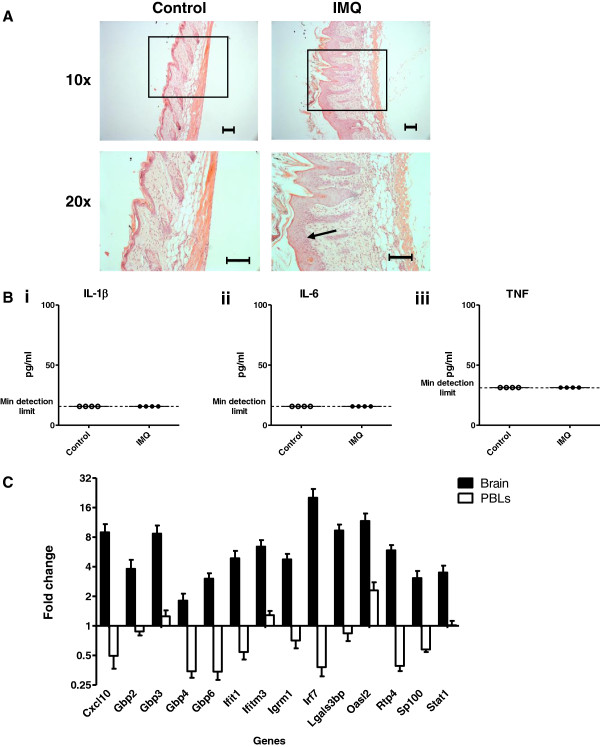
**Response in the brain to IMQ-induced skin inflammation.** Mice were treated daily with IMQ or control cream (Vaseline). Samples taken 24 hours after the fifth application. **(A)** H & E stains of (i) IMQ-treated and (ii) Vaseline-treated skin. Scale bar: 100 μm. Arrow points to epidermal hyperplasia. **(B)** Plasma concentrations of (i) IL-1β, (ii) IL-6 and (iii) TNFα. **(C)** Relative expression of ISGs, normalized to TBP, in brain and PBLs of IMQ-treated compared with Vaseline-treated mice. Fold change was calculated by comparing the normalized copy number in each sample to the mean of the vehicle-injected control group. Data represent mean ± SEM; *n* = 5/group.

Our data demonstrate that following acute or chronic LPS challenge, the response in the brain culminates in the transcription of a panel of ISGs. To determine whether TLR-induced inflammation could induce ISG expression in a tissue-specific peripheral, but not systemic, model of inflammation, we decided to assess the expression of target ISGs in mouse brains obtained following topical, cutaneous administration of a TLR7 agonist. Mice were challenged daily with topical applications of Imiquimod (IMQ) cream on their dorsal skin for five consecutive days and sacrificed for tissue harvesting 24 hours later. This is a standard method used to create a psoriasis-like pathology [15]. After two applications, all IMQ-treated mice showed signs of psoriasis-like skin inflammation, characterized by skin thickening, erythema and scaling. The severity of the symptoms peaked after the fourth application (data not shown). Figure 7A shows representative histological images of the skin inflammation present after five applications of IMQ or control cream. Like human psoriasis lesions, dorsal skin samples from IMQ-treated mice display epidermal hyperplasia. No signs of inflammation were observed following control cream (Vaseline)-treatment. The concentration of circulating IL-1β, IL-6 and TNFα was measured by ELISA. No significant increase in circulating cytokines was detected 24 hours after the final application of IMQ (Figure 7B). Thus, daily applications of IMQ induce a psoriasis-like skin pathology that causes local, but not systemic, inflammation.

At 24 hours after the final application, all target ISGs were significantly upregulated in the brains of IMQ-treated mice (Figure 7C, Table 3). Importantly, these data identify a common transcriptional signature in the brains of LPS- and IMQ-treated mice. Strikingly, in contrast with the brain, ISG expression in the PBLs did not significantly deviate from baseline. The difference in ISG regulation in the brain and PBLs following IMQ-treatment again highlights a brain-specific response to peripheral inflammation that does not appear to be mediated by circulating inflammatory cytokines.

**Table 3:** Significance of differentially expressed target genes following chronic inflammatory models

**Gene**	**Significance of fold change in treatment group compared to vehicle control group (↑, upregulated; ↓, downregulated)**			
	**IMQ (*P*)**		**TPA (*P*)**	
	**Brain**	**PBLs**	**Brain**	**PBLs**
***Ctsc***	↑ 0.0187	Not significant	↓ 0.0026	Not significant
***Cxcl10***	↑ 0.0156	Not significant	Not significant	Not significant
***Gbp2***	↑ 0.0177	Not significant	↓ 0.0401	Not significant
***Gbp3***	↑ 0.0032	Not significant	Not significant	Not significant
***Gbp4***	↑ 0.0254	Not significant	Not significant	↓ 0.0490
***Ifit1***	↑ 0.0053	Not significant	Not significant	Not significant
***Ifitm3***	↑ 0.0010	Not significant	Not significant	Not significant
***Igrm1***	↑ 0.0005	Not significant	Not significant	Not significant
***Irf7***	↑ 0.0029	Not significant	Not significant	Not significant
***Lgals3bp***	↑ 0.0003	Not significant	Not significant	Not significant
***Oasl2***	↑ 0.0015	Not significant	Not significant	Not significant
***Rtp4***	↑ 0.0002	Not significant	Not significant	Not significant
***Sp100***	↑ 0.0087	Not significant	Not significant	Not significant
***Stat1***	↑ 0.0043	Not significant	↓ 0.0241	Not significant

Significance of differential gene expression was calculated separately for individual genes in each tissue using an unpaired *t* test.

### Lack of ISG induction following sterile psoriasis-like skin inflammation

**Figure 8:**
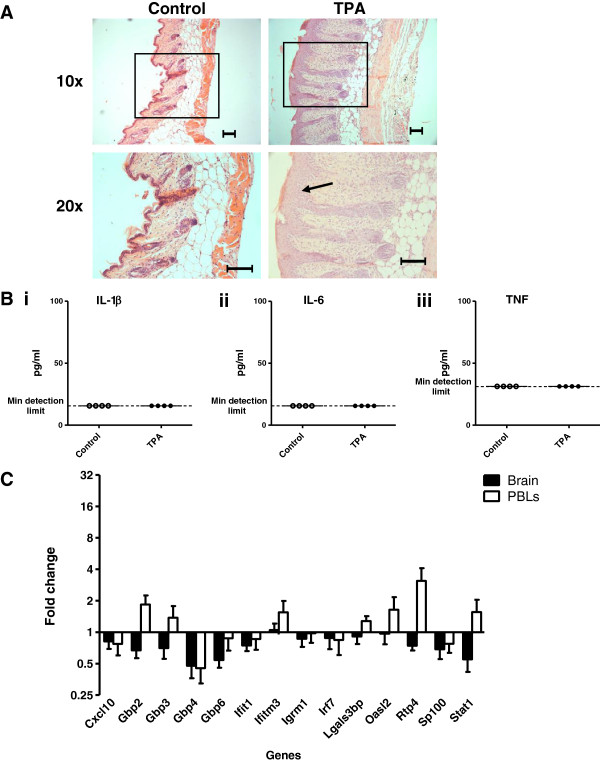
**Response in the brain to TPA-induced skin inflammation.** Mice were treated daily with five topical applications of TPA or acetone. Samples were taken 24 hours after fifth application. **(A)** H & E stains of (i) TPA-treated and (ii) acetone-treated skin. Scale bar: 100 μm. Arrow points to epidermal hyperplasia. **(B)** Plasma concentrations of (i) IL-1β, (ii) IL-6 and (iii) TNFα. **(C)** Relative expression of ISGs, normalized to TBP, in brain and PBLs of TPA-treated compared to acetone-treated mice. Fold change was calculated by comparing the normalized copy number in each sample to the mean of the vehicle-injected control group. Data represent mean ± SEM; *n* = 5/group.

Central ISG expression was also measured in a sterile model of skin inflammation to determine the importance of TLR ligation. Mice were challenged daily with topical applications of 12-O-tetradecanoylphorbol-13-acetate (TPA) on their dorsal skin for five consecutive days. Like human psoriasis and IMQ-treatment, TPA is thought to induce skin inflammation in an IL-17 and IL-23-dependent manner [32]. However, unlike IMQ, TPA does not result in TLR ligation. After two applications, all TPA-treated mice displayed psoriasis-like skin thickening, lesioning and erythema. Symptoms gradually worsened until the end of the model (data not shown). Figure 8A shows histological images of the skin inflammation present after five applications of TPA or vehicle. Like those from the IMQ model, dorsal skin samples taken from TPA-treated mice displayed epidermal hyperplasia. Only mild signs of inflammation were observed when mice were treated with the vehicle. At 24 hours after the final application, no inflammatory cytokines were detected in the circulation (Figure 8B). Therefore, topical applications of TPA induce a psoriasis-like pathology with similar characteristics to the inflammatory skin condition induced by IMQ.

Despite both the IMQ- and TPA-treated mice displaying similar skin pathology, no upregulated ISG expression was detected in the brains of the TPA-treated mice compared with the vehicle-treated control group (Figure 8C, Table 3). This is in stark contrast to the significant induction of all target ISGs that was detected in the brains of the IMQ-treated group. *Gbp4* was the only ISG to be significantly upregulated in the PBL. The lack of response in the brain following TPA-treatment suggests that ISG induction in the brains of IMQ-treated mice is not a generic effect of skin inflammation.

## Discussion

Here we demonstrate that systemic administration of LPS or topical administration of IMQ alters the gene expression profile in the brain, inducing the expression of a common panel of ISGs. Differing in kinetics and magnitude, this response is distinct from that of the PBLs. We also highlight ISG induction as a consequence of a TLR-induced type I IFN response. As considerable evidence links type I IFNs to psychiatric disorders [8-12], IFN production in the brain may represent a significant mechanism, linking peripheral TLR-induced inflammation with neuropsychiatric symptoms.

The differential response of brain and PBLs provides evidence of a brain-specific inflammatory response resulting from both acute and chronic LPS challenge and IMQ-induced skin inflammation. This was not a downstream by-product of peripheral inflammatory cytokine production as similar sterile models of inflammation failed to induce the same response. Furthermore, ISG expression remained elevated in the brain following daily LPS or IMQ administration, long after the peripheral cytokine response was attenuated. Thus, ISG induction was brain-specific and not mediated by peripheral inflammatory cytokines in either the skin or the circulation.

As type I IFN production is a classic hallmark of both TLR4-induced IRF3 activation and TLR7-induced IRF7 activation, it would be attractive to propose that a TLR-induced IFN response is responsible for the induction of ISGs in the brain. Supporting this hypothesis, Skelly and colleagues documented a central induction of IFNβ within 2 hours of systemic LPS challenge [33]. No central or peripheral type I IFN induction was observed following systemic TNFα or IL-1β injection. Furthermore, Leung and colleagues demonstrated that, following systemic administration of a TLR7 agonist, IFNα was induced in the brain in an IRF7-dependent manner [34]. Both TLR4 and TLR7 are widely expressed in the brain. Although, to our knowledge, the capacity of IMQ to cross the blood–brain barrier (BBB) has not been investigated, a study with radiolabelled LPS suggested that negligible levels cross the intact BBB [35]. Therefore, it is possible that the upregulation of target ISGs, following peripheral LPS or IMQ challenge, occurs via an indirect route downstream of IRF activation in the periphery. This may involve the direct action of peripherally produced IFNs on the CNS, the BBB or afferent nerves. Alternatively, introducing high or repeated doses of LPS or IMQ to the periphery may cause BBB breakdown, facilitating the direct action of these TLR ligands on the brain.

To further investigate the involvement of IRF activation in the neuroinflammation induced by systemic LPS injection, mice were challenged with the TLR2 ligand LTA. Consistent with previous reports [36], no response was detectable in the brains of mice following peripheral LTA injection. This lack of response may be due to the inability of TLR2 ligands to stimulate IRF-dependent signalling. Conversely, peripheral stimulation with TLR3 ligands is known to trigger brain inflammation [37,38]. Like the MyD88-independent pathway downstream of TLR4, TLR3 signals through the adaptor molecule TRIF to activate IRF3, ultimately triggering IFNβ production [31]. Subsequently, it would appear that IRF-dependent signalling, whether it occurs in the periphery or the brain, may be a requirement of ISG induction in the brain following systemic administration of TLR ligands.

As described, type I IFN therapy is intrinsically linked to severe neuropsychiatric disorders, mainly major depression [8-12]. It is well known that injecting rodents with LPS initiates a number of behavioural adaptations, including a depression-like behaviour that perseveres after the other sickness behaviours have resolved [1]. A recent report has also linked IMQ-treatment to the development of sickness behaviours in rats [39]. The elevated transcription of type I ISGs in the brain following LPS or IMQ challenge is a strong indication that type I IFNs are produced during these models. As this family of cytokines are well known for their effects on behaviour, type I IFN production in either the periphery or in the brain, following peripheral LPS or IMQ challenge could contribute to the onset of depression-like behaviours in rodents. Formal demonstration of this will require the use of rodent behavioural models in conjunction with appropriate gene-deficient mice.

## Conclusions

Toll-like receptor ligands have the capacity to modulate ISG expression distally in a manner that may be dependent on TLR-induced type I IFN production. Whether type I IFNs are produced in the brain or whether peripherally induced IFNs directly access the brain to modulate ISG expression remains open to further investigation; as does the downstream effects of central ISG induction. Owing to the well-established link between type I IFNs and depression, TLR-induced IFN production is worth investigating as a potential key mechanism, linking peripheral inflammation with sickness behaviour.

## Abbreviations

ANOVA: analysis of variance; BBB: blood–brain barrier; CNS: central nervous system; cPRR: cytosolic pattern recognition receptor; DAVID: Database for Annotation, Visualization and Integrated discovery; ELISA: enzyme-linked immunosorbent assay; GBP: guanylate-binding protein; H & E: haematoxylin and eosin; IFN: interferon; IL: interleukin; IMQ: Imiquimod; i.p.: intraperitoneally; IRF: interferon regulatory factor; ISG: interferon-stimulated gene; i.v.: intravenously; IκB: NFκB inhibitor; LPS: lipopolysaccharide; LTA: lipoteichoic acid; NFκB: nuclear factor κ-light-chain-enhancer of activated B cells; PBL: peripheral blood leucocyte; PBS: phosphate-buffered saline; qPCR: quantitative polymerase chain reaction; RMA: robust multichip average; SEM: standard error of the mean; TBP: TATA binding protein; TIR: Toll/interleukin-1 receptor; TLR: toll-like receptor; TNF: tumour necrosis factor; TPA: 12-O-tetradecanoylphorbol-13-acetate; TRIF: domain-containing adaptor protein.

## Competing interests

The authors declare that they have no competing interests.

## Authors’ contributions

All data was collected, analyzed and interpreted by CT and AM. Experiments were conceived and designed by JC and GG. The manuscript was drafted by CT and all authors read and approved the final manuscript.

## Supplementary Material

Additional file 1: Table S1 Primer sequences. Click here for file

Additional file 2: Table S2 Analysis of differentially expressed entities identified using Partek. Click here for file

Additional file 3: Table S3 Analysis of differentially expressed entities identified using GeneSpring. Click here for file
