# Supplementary material for: Peripheral inflammation is associated with remote global gene expression changes in the brain
Source: J Neuroinflammation. 2014 Apr 8;11:73. doi: 10.1186/1742-2094-11-73 (PMC4022192; doi:10.1186/1742-2094-11-73)
Supplement: Additional file 1: Table S1 — Primer sequences. [file 1742-2094-11-73-S1.pdf]

Additional file 1: Table S1. Primer Sequences

Gene Symbol	Primer Sequences (5' – 3')	
**Ctsc**	Forward	AGG CCA CAC AGC TAT CAG TT
	Reverse	TTG CCA ACA AAG CAA GCC CA
**Cxcl10**	Forward	GCT CAA GTG GCT GGG ATG
	Reverse	GAG GAC AAG GAG GGT GTG G
**Gbp2**	Forward	CCA AGC GAG ATG CCT TTA TC
	Reverse	TTC TTC TTC CAG GGG TCC A
**Gbp3**	Forward	TGC TTT GTC TTT GAC AGG CCC A
	Reverse	TCT TGG TCT TGC CGT TGG TGA A
**Gbp4**	Forward	CCT CTT CCT CTT TCT TCT TCC TTT
	Reverse	GTG TTT CTA TGG GGG TGT GG
**Ifit1**	Forward	GAC AAG GCA ATC ACC CTC TAC T
	Reverse	TCT TTC AGC CAC TTT CTC CAA
**Ifitm3**	Forward	CGC TCC ATC CTT TGC CCT TCA GTG
	Reverse	GCC CCC ATC TCA GCC ACC TCA T
**Il1b**	Forward	CGC TCA GGG TCA CAA GAA AC
	Reverse	GAG GCA AGG AGG AAA ACA CA
**Il6**	Forward	TTC CAT CCA GTT GCC TTC TT
	Reverse	ATT TCC ACG ATT TCC CAG AG
**Irgm1**	Forward	AGT TCA GCA GGT AGC CCA GA
	Reverse	TCA GCC TCA GTT TCC AGT CC
**Irf7**	Forward	GAA GAG GCT GGA AGA CCA ACT
	Reverse	AGA TAA AAC GCC CTG TGC TG

**Lgals3bp**	Forward	ATT CCT GTG TCC CCT CCT TC
	Reverse	GTG AGT GCT GGC TGA AAC CT
**Oasl2**	Forward	AGC GAG CGA GGG ATG TTC AGG T
	Reverse	TGG GGC TGT AGG GGT TTG TCC AG
**Rtp4**	Forward	GCA TCT TTG GGT GAG AAG GT
	Reverse	ATG GGG AGG AAC TCT TTG GT
**Sp100**	Forward	CAT CAT TTT CCT TGG CTG GT
	Reverse	CAT TTT GGT TGG TCC TTG CT
**Stat1**	Forward	GAA AAA CGC TGG GAA CAG AA
	Reverse	CGA CAG GAA GAG AGG TGG TC
**Tbp**	Forward	TGC TGT TGG TGA TTG TTG GT
	Reverse	AAC TGG CTT GTG TGG GAA AG
**Tnfa**	Forward	CAC CAC CAT CAA GGA CTC AA
	Reverse	GAG GCA ACC TGA CCA CTC TC
