# Supplementary material for: Peripheral inflammation is associated with remote global gene expression changes in the brain
Source: J Neuroinflammation. 2014 Apr 8;11:73. doi: 10.1186/1742-2094-11-73 (PMC4022192; doi:10.1186/1742-2094-11-73)
Supplement: Additional file 2: Table S2 — Analysis of differentially expressed entities identified using Partek. [file 1742-2094-11-73-S2.pdf]

Additional file 2: Table S2. Analysis of differentially expressed entities identified using Partek

Genbank Accession	Gene Symbol	Gene Name	Fold Change	P-value
**Fold Change ≥ 2**				
NM_008491	Lcn2	Lipocalin 2	29.29	2.61E-05
NM_011315	Saa3	Serum amyloid A3	11.60	2.56E-04
NM_016850	Irf7	Interferon regulatory factor 7	6.08	1.1E-04
NM_011854	Oasl2	2'-5' Oligoadenylate synthetase-like 2	5.44	1.01E-04
BC150711	AI607873	Interferon activated gene 204 homologue	4.93	3.74E-05
NM_023386	Rtp4	Receptor transporter protein 4	4.75	1.21E-04
NM_025378	Ifitm3	Interferon induced transmembrane protein	4.22	1.30E-05
NM_011579	Tgtp1	T-cell specific GTPase 1	3.79	3.64E-04
NM_008620	Gbp4	Guanylate binding protein 4	3.76	1.39E-04
NM_013563	Il2rg	Interleukin 2 receptor, gamma chain	3.71	9.29E-05
NM_023065	Ifi30	Interferon gamma inducible protein 30	3.64	9.95E-05
NM_144559	Fcgr4	Fc receptor, IgG, low affinity IV	3.57	4.23E-06
NM_194336	Gbp6	Guanylate binding protein 6	3.54	5.68E-06

NM_009252	Serpina3n	Serine (or cysteine) peptidase inhibitor, clade A	3.52	3.12E-04
NM_001082960	Itgam	Integrin alpha M	3.41	2.45E-04
NM_001033767	Gm4951	Predicted gene 4951	3.36	4.14E-05
---	N/A*	---	3.35	2.71E-05
NM_011150	Lgals3bp	Lectin, galactoside-binding, soluble, 3 binding protein	3.31	7.93E-05
NM_001001892	H2-K1	Histocompatibility 2, K1, K region	3.24	3.11E-04
NM_008331	Ifit1	Interferon-induced protein with tetratricopeptide repeats	3.23	3.50E-05
NM_009780	C4b	Complement component 4B (Childo blood group)	3.13	2.78E-04
NM_011905	Tlr2	Toll-like receptor 2	3.09	1.74E-05
---	Rnf213**	Ring finger protein 213	3.09	1.22E-04
AK173199	Rnf213	Ring finger protein 213	3.07	5.21E-06
NM_010260	Gbp2	Guanylate binding protein 2	3.02	8.62E-05
NM_013673	Sp100	Nuclear antigen Sp100	3.02	1.8E-04
NM_145545	Gbp7	Guanylate binding protein 7	2.86	3.04E-05
---	Rnf213**	Ring finger protein 213	2.74	1.15E-04
NM_010130	Emr1	EGF-like module containing, mucin-like, hormone receptor-li	2.72	1.74E-04
---	Rnf213	Ring finger protein 213	2.69	1E-04
---	N/A*	---	2.69	2E-04

NM_011693	Vcam1	Vascular cell adhesion molecule 1	2.68	1.11E-04
NM_018734	Gbp3	Guanylate binding protein 3	2.66	6.62E-05
NM_009402	Pglyrp1	Peptidoglycan recognition protein 1	2.56	1.89E-05
NM_010738	Ly6a	Lymphocyte antigen 6 complex, locus A	2.55	3.80E-05
NM_009283	Stat1	Signal transducer and activator of transcription 1	2.51	2.41E-04
NM_009982	Ctsc	Cathepsin C	2.47	3.09E-06
NM_153197	Clec4a3	C-type lectin domain family 4, member a3	2.46	2.91E-04
AK173199	Rnf213	Ring finger protein 213	2.39	2.53E-04
NM_008879	Lcp1	Lymphocyte cytosolic protein 1	2.36	1.27E-04
NM_008326	Irgm	immunity-related GTPase family M member 1	2.33	3.25E-04
---	N/A*	---	2.32	1.42E-04
NM_001160415	Apobec3	apolipoprotein B mRNA editing enzyme	2.32	1.86E-04
NM_031195	Msr1	Macrophage scavenger receptor 1	2.29	4.58E-05
---	Rnf213**	Ring finger protein 213	2.26	1.29E-05
NM_011708	Vwf	Von Willebrand factor homolog	2.26	3.89E-05
NM_031376	Pik3ap1	phosphoinositide-3-kinase adaptor protein 1	2.21	1.41E-04
NM_021384	Rsad2	Radical S-adenosyl methionine domain containing 2	2.18	2.37E-04

---	N/A*	---	2.13	1.4E-04
NM_001163522	Emcn	Endomucin	2.12	1.34E-04
NM_001037713	Xaf1	XIAP associated factor 1	2.09	1.74E-04
NM_001113356	C1rb	Complement component 1, r subcomponent B	2.09	1.99E-04
NM_013690	Tek	Endothelial-specific receptor tyrosine kinase	2.06	1.37E-04
NM_012054	Aoah	Acyloxyacyl hydrolase	2.04	3.06E-04
NM_001039530	Parp14	Poly (ADP-ribose) polymerase family, member 14	2.02	7.28E-05
**Fold Change ≥ 1.5**				
NM_013805	Cldn5	claudin 5	1.97597	8.43E-05
---	Rnf213**	Ring finger protein 213	1.91996	1.46E-04
NM_172479	Slc38a5	Solute carrier family 38, member 5	1.86936	8.83E-05
NM_001081215	Ddx60	DEAD (Asp-Glu-Ala-Asp) box polypeptide 60	1.8692	1.33E-05
NM_010833	Msn	Moesin	1.85669	1.59E-04
NM_010741	Ly6c1	lymphocyte antigen 6 complex, locus C1	1.84075	1.33E-04
NR_030719	Gm8979	very large inducible GTPase 1 pseudogene	1.83413	8.11E-05
BC023105	BC023105	cDNA sequence BC023105	1.81633	5.22E-05
NM_025992	Herc5	hect domain and RLD 5	1.80396	2.4E-04
NM_009868	Cdh5	cadherin 5	1.80082	1.62E-05

NM_010493	Icam1	intercellular adhesion molecule 1	1.79505	1.36E-04
---	N/A*	---	1.76818	1.63E-04
---	Rnf213**	Ring finger protein 213	1.763	3E-04
NR_003507	Oas1b	2'-5' oligoadenylate synthetase 1B	1.75542	1.55E-04
NM_010104	Edn1	endothelin 1	1.74503	1.66E-04
NM_028261	Tmem173	transmembrane protein 173	1.73832	2.27E-05
NM_010225	Foxf2	forkhead box F2	1.71563	7.39E-05
NM_007609	Casp4	caspase 4, apoptosis-related cysteine peptidase	1.70807	1.29E-04
NM_031181	Siglece	sialic acid binding Ig-like lectin E	1.69006	1.7E-04
NM_009888	Cfh	complement component factor h	1.64032	2.28E-04
NM_001037298	Fam38a	family with sequence similarity 38, member A	1.62396	9.95E-05
NM_019963	Stat2	signal transducer and activator of transcription 2	1.60841	1.01E-04
NM_030253	Parp9	poly (ADP-ribose) polymerase family, member 9	1.59804	1.17E-04
NM_001111059	Cd34	CD34 antigen	1.59774	8.42E-05
---	N/A*	---	1.58439	1.27E-04
NM_025659	Abi3	ABI gene family, member 3	1.54831	1.42E-04
---	N/A*	---	1.52005	3.61E-04
NM_028195	Cyth4	cytohesin 4	1.5195	1.00E-04
NM_183168	P2ry6	pyrimidinergic receptor P2Y, G-protein coupled, 6	1.51848	3.49E-04
NM_007705	Cirbp	cold inducible RNA binding	-1.5643	3.09E-04

protein

Significance was calculated using one-way ANOVA and a Benjamini-Hochberg multiple testing correction.

*Probe sets don’t map to annotated genes

**Annotations manually determined using Affymetrix online database, NetAffx
