# Supplementary material for: Peripheral inflammation is associated with remote global gene expression changes in the brain
Source: J Neuroinflammation. 2014 Apr 8;11:73. doi: 10.1186/1742-2094-11-73 (PMC4022192; doi:10.1186/1742-2094-11-73)
Supplement: Additional file 3: Table S3 — Analysis of differentially expressed entities identified using GeneSpring. [file 1742-2094-11-73-S3.pdf]

Additional file 3: Table S3. Analysis of differentially expressed entities identified using GeneSpring

Genbank Accession	Gene Symbol	Gene Name	Fold Change	P-value
NM_008491	Lcn2	Lipocalin 2	16.58	8.27E-06
NM_011315	Saa3	Serum amyloid A3	8.69	1.91E-04
BC150711	AI607873	Interferon activated gene 204 homologue	3.93	7.84E-05
NM_011854	Oasl2	2'-5' Oligoadenylate synthetase-like 2	3.91	1.25E-04
NM_025378	Ifitm3	Interferon induced transmembrane protein	3.69	3.91E-05
NM_009252	Serpina3n	Serine (or cysteine) peptidase inhibitor, clade A	3.32	1.65E-04
NM_009099	Trim30	Tripartite motif-containing 30	3.28	9.56E-05
NM_008331	Ifit1	Interferon-induced protein with tetratricopeptide repeats	3.12	1.05E-04
NM_008329	Ifi204	Interferon activated gene 204	3.05	6.68E-05
NM_133871	Ifi44	Interferon activated gene 44	2.94	1.44E-04
NM_023386	Rtp4	Receptor transporter protein 4	2.93	4.51E-05
NM_021274	Cxcl10	chemokine (C-X-C motif) ligand 10	2.91	8.88E-05
NM_144559	Fcgr4	Fc receptor, IgG, low affinity IV	2.90	2.18E-05
NM_011150	Lgals3bp	Lectin, galactoside-binding, soluble, 3 binding protein	2.89	5.40E-05

NM_194336	Gbp6	Guanylate binding protein 6	2.70	4.41E-06
NM_013563	Il2rg	Interleukin 2 receptor, gamma chain	2.64	4.41E-06
---	N/A*		2.60	4.42E-05
NM_001001892	H2-K1	Histocompatibility 2, K1, K region	2.49	4.42E-05
NM_010260	Gbp2	Guanylate binding protein 2	2.42	1.13E-04
NM_028595	Ms4a6c	Membrane-spanning 4-domains, subfamily A, member 6C	2.40	1.91E-04
AK173199	Rnf213|LOC672511	Ring finger protein 213	2.34	5.92E-07
NM_009402	Pglyrp1	Peptidoglycan recognition protein 1	2.36	8.03E-05
---	Rnf213**	Ring finger protein 213	2.33	1.36E-05
NM_009982	Ctsc	Cathepsin C	2.31	5.42E-05
NM_010738	Ly6a	Lymphocyte antigen 6 complex, locus A	2.29	1.60E-05
NM_018734	Gbp3	Guanylate binding protein 3	2.28	1.60E-04
NM_172689	Ddx58	DEAD box polypeptide 58	2.27	1.26E-04
NM_145545	Gbp7	Guanylate binding protein 7	2.27	3.80E-06
NM_009780	C4b |C4a	complement component 4B (Childo blood group) | complement 4A (Rodgers blood group)	2.24	1.40E-04

NM_013673	Sp100	Nuclear antigen Sp100	2.22	4.61E-05
NM_026835	Ms4a6d	Membrane-spanning 4-domains, subfamily A, member 6D	2.21	4.36E-05
NM_008326	Irgm	Immunity-related GTPase family M member 1	2.21	2.56E-05
NM_013805	Cldn5	Claudin 5	2.10	1.42E-04
---	Rnf213**	Ring finger protein 213	2.08	2.78E-05
NM_199146	AI451617	Expressed sequence AI451617	2.06	8.85E-05
NM_009283	Stat1	Signal transducer and activator of transcription 1	2.04	5.75E-06
---	Rnf213**	Ring finger protein 213	2.02	2.69E-06
NM_153564	GBP5	Guanylate binding protein 5	2.02	5.17E-05
NM_001160415	Apobec3	Apolipoprotein B mRNA editing enzyme	2.02	2.07E-05

Significance was calculated using an unpaired t-test and a Benjamini-Hochberg multiple testing correction.

*Probe sets don’t map to annotated genes

**Annotations manually determined using Affymetrix online database, NetAffx
